# The Use of Fiberoptic Bronchoscopy During Percutaneous Dilatational Tracheostomy With Laryngeal Mask

**DOI:** 10.1155/DTE.4.13

**Published:** 1997

**Authors:** Maurizio Rossi, Marco De Monti, Davide Sonnino, Bruno Giacometti

**Affiliations:** 1 Intensive Care Unit General Hospital of Menaggio Como 22017 Italy; 2 S.S.U.E.m.118 (Italian Emergency Medical Service) of Menaggio Como 22017 Italy; 3 Department of General Surgery General Hospital of Menaggio Como 22017 Italy; 4 Via Derna 5 Milano I-20132 Italy

## Abstract

The aim of our research is to evaluate the advantage by the combined use of fiberoptic bronchoscopy and laryngeal mask during the performance of percutaneous dilatational tracheostomy in an intensive care unit.

*Patients*: 16 adult patients who were candidates to middle-long term mechanical ventilation.

*Environment*: Intensive Care Unit of a Community General Hospital.

*Results*: We experienced 3 minor complications (2 minor bleedings and 1 neck emphysema). Difficulties were found in 3 patients with particular anatomical conformation (obese patients with short neck and limited mobility of the cervical spine).

*Conclusion*: The combined use of fiberoptic tracheo-bronchoscopy with the laryngeal mask permits a better endoscopic visualisation of the operatory field, providing a more secure and precise procedure.


*Diagnostic and Therapeutic Endoscopy*, Vol. 4, pp. 13–18
Reprints available directly from the publisher
Photocopying permitted by license only

© 1997 OPA (Overseas Publishers Association)
Amsterdam B.V. Published in The Netherlands
by Harwood Academic Publishers
Printed in Singapore

### The Use of Fiberoptic Bronchoscopy During Percutaneous Dilatational Tracheostomy with Laryngeal Mask

MAURIZIO ROSSI[a], MARCO DE MONTI[b,*], DAVIDE SONNINO[c] and BRUNO GIACOMETTI[a]

[a]*Intensive Care Unit, General Hospital of Menaggio, 22017 (Como), Italy;* [b]*S.S.U.E.m.118 (Italian Emergency Medical Service) of Menaggio, 22017 (Como), Italy;* [c]*Department of General Surgery, General Hospital of Menaggio, 22017 (Como), Italy*

*(Received 18 September 1996; Revised 12 February 1997; In final form 27 February 1997)*

**The aim of our research is to evaluate the advantage by the combined use of fiberoptic bronchoscopy and laryngeal mask during the performance of percutaneous dilatational tracheostomy in an intensive care unit.**

***Patients*: 16 adult patients who were candidates to middle-long term mechanical ventilation.**

***Environment*: Intensive Care Unit of a Community General Hospital.**

***Results*: We experienced 3 minor complications (2 minor bleedings and 1 neck emphysema). Difficulties were found in 3 patients with particular anatomical conformation (obese patients with short neck and limited mobility of the cervical spine).**

***Conclusion*: The combined use of fiberoptic tracheo-bronchoscopy with the laryngeal mask permits a better endoscopic visualisation of the operatory field, providing a more secure and precise procedure.**

*Keywords:* Percutaneous dilatational tracheostomy, Fiberoptic tracheo-bronchoscopy, Laryngeal mask

### INTRODUCTION

Methods of percutaneous dilatational tracheostomy, originally described by Ciaglia in 1985 [1], and similar techniques, are having a wide employment in intensive care in comparison with the classic surgical tracheostomy. The global incidence of complications in the scientific literature for the classic technique is about 22% [2–8]. Complications mainly include: stomal infections, stomal granulomas, major bleedings, and subcutaneous emphysema.

However, employing the percutaneous technique, these same complications only reach a 12% global incidence, with a greatly reduced rate of stomal infections. Additionally, the percutaneous tracheotomy has some significant advantages such as:

(a) feasibility of the operation at the patient's bedside in the Intensive Care Unit;

* Corresponding author. Via Derna 5, I-20132 Milano, ITALY. Tel.: +39 2 26 11 13 44, +39 330 23 05 55. Fax: +39 344 30596.

(b) low learning curve due to ease of procedure;
(c) operatory room cost savings;
(d) short length of procedure.

The introduction of endoscopic guides, first suggested by Marelli and associated in 1990 [9,10] and followed by other Authors [11], represented an improvement of the original technique and caused a further reduction of complications related to tracheostomy.

In fact, endoscopic vision permits a precise visualisation of the dilators and tracheostomy tube, and allows an immediate correction of improper accesses or procedures such as paramedian needle insertion.

In our experience with the fiberoptic bronchoscope we also employed the laryngeal mask, as previously described by Dexter [12] and Tarpey [13], in order to reduce several disadvantages associated with the classic procedure, which included difficulty in maintaining the endotracheal tube in the correct position, and problems related to the relative pressure of the endotracheal tube cuff, positioned immediately proximal to the vocal cords, on the larynx and on the same vocal cords, sometimes damaging these structures. The laryngeal mask permits, indeed, a more clear endoscopic vision of the operatory field in comparison to introducing the scope through the endotracheal tube.

### PATIENTS AND METHODS

From March 1995 to March 1996 in the Intensive Care Unit of the Community General Hospital of Menaggio (Como) 16 percutaneous dilatational tracheostomy were performed in patients candidate to middle-long term mechanical ventilation. Data regarding our series are shown in Table I.

Of the 16 patients included in our series, 9 were females (56%) and 7 were males (44%), the average age was 77, ranging between 65 and 93 years old. The SAPS II average value was 49.9, ranging between 21 and 75.

The global mortality rate during the hospital stay was 56%, out of which 37.5% (6 patients) in the Intensive Care Unit. However, no death was related to complications due to percutaneous dilatational tracheostomy.

The following pathologies were observed: 10 patients were affected by reacute chronic obstructive bronchopneumonopathy, with pneumonia in 5 of these patients; 2 patients were affected by intraparenchymatous brain haemorrhage; 3 patients were affected by a septic state caused by surgical pathologies (perforated gastric ulcer; liver carcinoma; perforated empyema of the gallbladder). The last patient had an acute respiratory failure due to a cardiogenic shock following an infarction. In average, we performed percutaneous dilatational tracheostomy on the 10th day of admittance, ranging between one and twenty days.

The average time of the operation was about 17 min, ranging from a minimum of 10 min for patients with favourable anatomical conditions to a maximum of about 25 min for obese patients with short and less mobile necks.

The percutaneous dilatational tracheostomy was performed at the bedside of the patient by two doctors of the Intensive Care Unit: one doctor performed the operation and the other managed the anaesthesia and particularly drugs, ventilation, and the correct positioning of the laryngeal mask. The procedure was also followed by two graduate nurses. The use of fiberoptic bronchoscope allowed everybody to follow the entire operation on the monitor, permitting a wide participation.

The operation was performed in intravenous general anaesthesia. Primary anaesthesia was obtained with propofol 2 mg/kg and fentanyl 0.05–0.1 mg; maintenance with propofol 1–3 mg/kg/h, pancuronium 0.8 mg/kg. Patients were ventilated via a Siemens 900 C until the laryngeal mask was positioned.

The ventilation was led in the following way: CPPV, TV of 7 ml/kg, $FiO2:1.0$.

TABLE I Summary of our series

| No. | Name | Sex | Age | SAPS II | Admittance Disease | Date of Admittance | Date of Tracheostomy | Duration of Procedure (min) | Complications | Notes |
|---|---|---|---|---|---|---|---|---|---|---|
| 1 | G.E. | M | 78 | 45 | Reacute coad | 03/02/95 | 03/06/95 | 20 | No | — |
| 2 | C.G. | M | 82 | 72 | Pneumonia | 03/11/95 | 03/15/95 | 15 | No | — |
| 3 | M.M. | F | 70 | 70 | Brain haemorrage | 03/19/95 | 03/23/95 | 25 | Minor bleeding | Tracheomalacia |
| 4 | B.M. | M | 78 | 75 | Pneumonia | 03/12/95 | 03/24/95 | 25 | No | — |
| 5 | P.F. | M | 78 | 72 | Reacute coad | 03/26/95 | 04/04/95 | 25 | No | Unfavourable anatomical conditions: Deviation of trachea for cyfoscholiosis – Improper placement of the guidewire |
| 6 | M.M. | F | 84 | 48 | Gallbladder empyema – Septic state | 05/04/95 | 05/16/95 | 15 | No | — |
| 7 | A.A. | M | 65 | 72 | AMI – Cardiogenic shock | 05/14/95 | 05/30/95 | 20 | No | — |
| 8 | C.B. | F | 68 | 60 | Brain haemorrage | 07/22/95 | 08/08/95 | 15 | No | — |
| 9 | V.M. | F | 69 | 21 | Reacute coad | 08/10/95 | 08/11/95 | 25 | No | Obese – Short neck: Tracheostomy between the cricoid cartilage and the first tracheal ring |
| 10 | D.D. | F | 85 | 35 | Perforated gastric ulcer – Septic state | 09/26/95 | 10/16/95 | 20 | No | Unfavourable anatomical conditions (short neck): Air leakage around the mask |
| 11 | P.R. | M | 68 | 27 | Liver carcinoma – Septic state | 10/26/95 | 11/06/95 | 15 | No | — |
| 12 | T.B. | F | 83 | 30 | Pneumonia | 12/31/95 | 01/06/96 | 10 | No | Unfavourable anatomical conditions (short neck): Air leakage around the mask |
| 13 | D.M. | F | 93 | 63 | Pneumonia | 01/14/96 | 01/18/96 | 10 | No | — |
| 14 | C.G. | F | 69 | 27 | Reacute coad | 01/04/96 | 01/18/96 | 15 | No | Obese – Short neck: Tracheostomy between the cricoid cartilage and the first tracheal ring |
| 15 | P.A. | F | 84 | 45 | Pneumonia | 03/14/96 | 03/26/96 | 20 | Minor bleeding + Subcutaneous neck emphysema | — |
| 16 | T.A. | M | 74 | 38 | Reacute coad | 03/16/96 | 03/28/96 | 20 | No | Unfavourable anatomical conditions (short neck): Air leakage around the mask |

We monitored blood's saturation by a pulse oximetry (DATEX SATLITE trans), non-invasive arterial pressure (COLIN PRESS-MATE BP 8800), and the electric cardiac activity (SOXIL SENTINEL 2A).

We used Ciaglia's kit for percutaneous tracheostomy (COOK CRITICAL CARE), a flexible fiberoptic bronchoscopy (Olympus BF type 3C30 OES Bronchofiberscope) and the laryngeal mask n.4 (INTAVENT).

Under the direct vision of the laryngoscopy, we initially suctioned the pharynx. The patient was then estubated and the laryngeal mask was positioned. In this phase, the patient was ventilated with 100% oxygen via a manual breathing unit. We introduced the fiberscope in order to obtain the transillumination of the interspace between the first and the second tracheal cartilages, which had been previously identified by palpation. Subsequently, percutaneous dilatational tracheostomy was performed following the schema described by Ciaglia [7]. The neck, partially hyperextended, is prepared with a disinfectant solution and the operatory field surrounded with sterile linen.

Cutis is infiltrated with lidocaine idrocloridate 1% with adrenaline (1:200.000) from the cricoid margin to the third tracheal ring, in order to avoid bleeding from cutaneous and subcutaneous layers. A 0.5–1 cm vertical incision of cutis and subcutis is performed, avoiding damage to blood vessels by transillumination of the bronchoscope.

A needle connected to a syringe filled with saline solution is inserted between the first and the second tracheal ring with a 35° cranio-caudal angle. The aspiration of air demonstrates the penetration of the needle in the trachea. A guidewire of 0.052 inch diameter with a "J" tip is inserted through the needle. Then a dilator and an 8-French Teflon guide are inserted on the

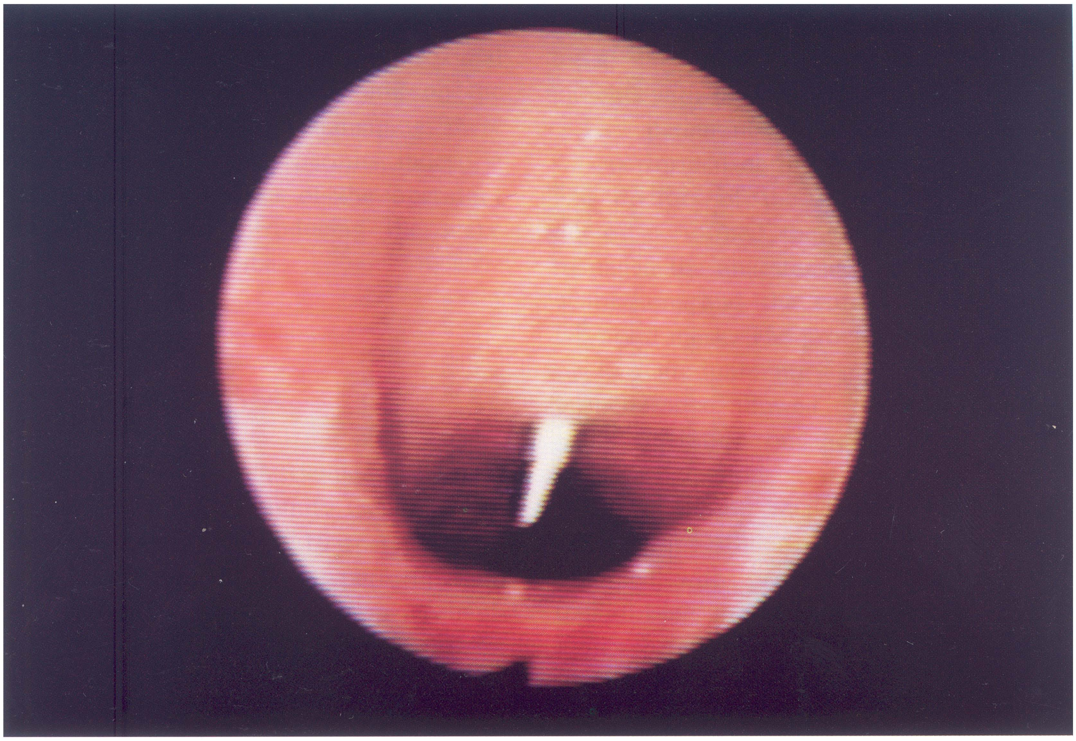

FIGURE 1 Bronchoscopical view through the laryngeal mask: the operatory field is good and it is clearly evident the 8 French Teflon guide inserted on the guidewire.

guidewire in order to make the system rigid (Fig. 1). The guide has a small ring on the distal end in order to avoid damages by the dilators, especially to the posterior wall of the trachea.

Next, dilators from 12 to 36 French are inserted on the guide.

When the required dilatation is obtained, a 7, 8, or 9 mm diameter tracheostomy tube is inserted, using a smaller dilator as a guide. Subsequently, the double guide is removed and the tube can be cuffed.

### RESULTS AND CONCLUSIONS

Data reported in literature, as previously described, demonstrates that most common complications following percutaneous tracheotomy include: subcutaneous emphysema, bleedings, stomal infections, difficulties in tracheostomy tube insertion and paramedian insertions. Global incidence of complications is about 12%. Our experience does not significantly differ from these results, and particularly, the complications which occurred in our patients are described in Table II.

We globally had 3 minor complications, and particularly two cases of minor bleeding stopped only by the compression obtained with the tracheostomy tube, and one case of subcutaneous emphysema of the neck which spontaneously healed in a few days.

By employing the fiberoptic bronchoscopy and the laryngeal mask we avoided incorrect introduction of the dilators, as we were able to correct any improper placement of the guidewire.

The difficulties we encountered always occurred in patients with unfavourable anatomical conditions (obese patients with short neck and limited mobility of the cervical spine), which do not appear ideally suited for this procedure.

In two of these patients, it was necessary to perform the tracheostomy between the cricoid cartilage and the first tracheal ring. In these two as well as one other patient, we found several difficulties in maintaining the laryngeal mask in the correct position, but were always able to complete the procedure.

Notwithstanding several other reports [12], in our series all patients had a nasogastric tube but we didn't have any noticeable leakage around the

TABLE II Complications and difficulties occurred during the procedure

| Complications | No. |
|---|---|
| Minor bleeding | 2 |
| Major bleeding (Blood transfusions) | No |
| Inflammatory infiltrate | No |
| Damage of posterior tracheal wall | No |
| Paramedian placement of the guidewire (Corrected under endoscopic vision) | (2) |
| Pneumotorax | No |
| Subcutaneous neck emphysema | 1 |
| Deaths related to the procedure | No |
| TOTAL | 3/16 (18%) |
| **Difficulties†** | **No.** |
| Tracheostomy performed between the cricoid cartilage and the first tracheal ring | 2 |
| Air leakage around the mask | 3 |
| TOTAL | 5/16 (31%) |

†Obese patients with short neck and limited mobility of the cervical spine.

laryngeal mask attributable to it; in three cases the mask leakage has been ascribed to unfavourable anatomical conditions (obesity and short neck).

Other Authors [14] evidenced that laryngeal mask could be dangerous due to the possibility of aspiration pneumonia: in our experience this evidence is not justified since we perform percutaneous dilatational tracheostomy only in elective patients and never in emergency, which is a different approach compared to other published literature [15].

We can conclude that the use of fiberoptic bronchoscopy with the laryngeal mask facilitates the performance of the Ciaglia's percutaneous dilatational tracheostomy. Particularly, endoscopic vision of the operatory field offered by laryngeal mask is greatly improved compared to insertion the instrument through the endotracheal tube.

Furthermore, the connection of the scope to a monitor allows the operator and his team to follow every step of the operation and perform the procedure with a greater safe and coordination.

### *References*

[1] Ciaglia, P., Firsching, R. and Syniec, C. Elective percutaneous dilational tracheostomy: a new simple bedside procedure; preliminary report. *Chest* 1985; **87**: 715–9.

[2] Rosi, R., De Gaudio, A.R. and Frova, G. Complicanze immediate delle tecniche di tracheostomia percutanea. *Anestes Rianim Intens* 1994; **15**: 316–22.

[3] Ciaglia, P. and Graniero, K.D. Percutaneous dilational tracheostomy: Results and long term follow up. *Chest* 1992; **101**: 464–7.

[4] Grigs, W.M., Myburgh, J.A. and Worthley, L.I. A prospective comparison of a percutaneous tracheostomy technique with standard surgical tracheostomy. *Intensive Care Med.* 1991; **17**: 261–3.

[5] Bodenham, A., Diament, R., Cohen, A. and Webster, N. Percutaneous dilational tracheostomy. A bedside procedure on the intensive care unit. *Anaesthesia* 1991; **46**: 570–2.

[6] Hazard, P., Jones, C. and Benitone, J. Comparative clinical trial of standard operative tracheostomy with percutaneous tracheostomy. *Crit. Care. Med.* 1991; **19**: 1018–24.

[7] Heffner, J.E., Miller, K.S. and Sahn, S.A. Tracheostomy in the intensive care unit. Part 2: Complications. *Chest* 1986; **90**: 430–6.

[8] Meade, J.W. Tracheostomy – its complications and their management – a study of 212 cases. *N. Eng. J. Med.* 1961; **265**: 519–523.

[9] Marelli, D., Paul, A., Manolidis, S., Walsh, G., Odim, J.N.K., Burdon, T.A., Shennib, H., Vestweber, K.H., Fleiszer, D.M. and Mulder, D.S. Endoscopic guided percutaneous tracheostomy: early results of a consecutive trial. *J. Trauma* 1990; **30**: 433–5.

[10] Paul, A., Marelli, D., Chiu, C.J., Vestweber, K.H. and Mulder, D.S. Percutaneous endoscopic tracheostomy. *Ann. Thoracic Surg.* 1989; **47**: 314–315.

[11] Winkler, W.B., Karnik, R., Seelmann, O., Havlicek, J. and Slany, J. Bedside percutaneous dilatational tracheostomy with endoscopic guidance: experience with 71 ICU patients. *Intensive Care Med.* 1994; **20**: 476–479.

[12] Dexter, T.J. The laryngeal mask airway: a method to improve visualisation of the trachea and larynx during fibreoptic assisted percutaneous tracheostomy. *Anaesth. Int. Care.* 1994; **22**: 35–39.

[13] Tarpey, J.J., Lynch, L. and Hart, S. The use of the laryngeal mask airway to facilitate the insertion of a percutaneous tracheostomy. *Intensive Care Med.* 1994; **20**: 448–449.

[14] Barker, P., Langton, J., Murphy, P. and Rowbotham, D. Regurgitation of gastric contents during general anaesthesia using the laryngeal mask airway. *Br. J. Anaesth.* 1992; **69**: 314–315.

[15] Lyons, B.J. and Flynn, C.G.M. The laryngeal mask simplifies airway management during percutaneous dilational tracheostomy. *Acta Anaesthesiol. Scand.* 1995; **39**: 414–415.

